# Anandamide Targets Membrane Integrity in Non-*Albicans Candida*: A Novel Antifungal Approach

**DOI:** 10.3390/jof12080616

**Published:** 2026-08-16

**Authors:** Goldie Wolfson, Doron Steinberg, Itzhack Polacheck, Maya Korem

**Affiliations:** 1 Institute of Biomedical and Oral Research (IBOR), Faculty of Dental Medicine, The Hebrew University of Jerusalem, Jerusalem 9112102, Israel; 2 Department of Clinical Microbiology and Infectious Diseases, Hadassah-Hebrew University Medical Center, Jerusalem 9112001, Israel

**Keywords:** Anandamide (AEA), non-*albicans Candida* (NAC) species, *Candidozyma* auris, *Candida glabrata*, *Candida parapsilosis*, membrane fluidity, efflux pump inhibition, ROS stress, antibiofilm activity, multidrug resistance

## Abstract

Fungal infections remain a major threat to human health, with non-*albicans Candida* (NAC) species causing more than half of all clinical cases and many strains gaining resistance to current treatments rapidly. Previously, N-arachidonoyl ethanolamine (anandamide, AEA) has been studied and shown to possess antibacterial and antifungal properties against various bacteria and *Candida albicans*. Given the previous findings, we aim here to expand the current preliminary research on AEA to investigate its antifungal activities against clinically relevant NAC species in vitro: *Candida glabrata*, *Candida parapsilosis*, and *Candidaozyma auris*. The minimum inhibitory concentration (MIC) and growth curve analysis determined planktonic inhibition. MTT metabolic assay and ATP production via BacTiter-Glo luminescence assay evaluated biofilm formation. Membrane fluidity, polarization and efflux pump activity were examined using fluorescence probes Laurdan, DiS-C3(3), and Rhodamine 6G, respectively. Reactive oxygen species (ROS) were assessed using DCFH-DA. Biofilm architecture and cell viability were analyzed by spinning disk confocal microscopy (SDCM). AEA reduced MIC values and slowed planktonic growth, while MTT and ATP assays demonstrated a pronounced dose-dependent reduction in biofilm metabolic activity. Membrane-targeted effects revealed increased fluidity and permeability at 125 µg/mL. Notably, AEA rapidly impaired efflux pump activity and induced intracellular ROS production. This effect was accompanied by reduced cell viability, increased proportions of PI-positive cells, and enhanced intracellular dye retention, as confirmed by SDCM. Together, these findings demonstrate that AEA exerts antifungal activity by disrupting membrane integrity and associated cellular functions and provide the first comparative characterization of species-specific membrane and oxidative stress responses to AEA across three major clinically relevant multidrug-resistant NAC species.

## 1. Introduction

Invasive candidiasis (IC) is a serious fungal infection caused by species of the genus *Candida*. Although more than 150 *Candida* species have been identified, only about 15 colonize humans as commensals and are capable of causing disease in both immunocompetent and immunocompromised individuals [1,2,3,4]. Despite the relatively small number of pathogenic *Candida* species, fungal infections remain one of the leading threats to human health with IC accounting for 8–10% of hospital-acquired bloodstream infections in the United States [5]. Previously, *Candida albicans* was the predominant cause of candidiasis; however, there has been a global shift toward non-*albicans Candida* (NAC) species such as *Candida glabrata*, *Candida parapsilosis* and *Candidozyma auris*, which now account for more than 60% of all cases [2,3,6,7,8]. This epidemiologic shift is driven by factors such as widespread use of antifungals, increased hospitalization rates, widespread invasive medical procedures and catheter use, parenteral nutrition, neutropenia, prior exposure to azoles, and increasing antifungal resistance [6,9]. *Candida* bloodstream and systemic infections carry a morbidity rate of 46–75% [7], and the growing prevalence of azole-resistant NAC strains further limits treatment options, posing a significant public health-threat [3].

Host predisposition risk factors and Candida virulence

Predisposing factors for candidiasis include prolonged antibiotic therapy, nutritional deficiencies, corticosteroid use, diabetes mellitus, immunosuppressive conditions and hormone therapy [10]. Once established, *Candida* species employ multiple virulence mechanisms, including adhesion to host tissues and abiotic surfaces, morphological transition from yeast to pseudo-hyphae and true hyphae, and formation of biofilms on mucosal surfaces and medical devices [11]. Fungal biofilms exhibit resistance to high levels of antifungals due to limited drug penetration through the extracellular matrix (EM), reduced growth rate, overexpression of drug resistance genes, particularly those encoding efflux pumps, and the presence of persistent tolerance cells [12]. As antifungal resistance against several drug classes continues to rise, the need for novel antibiofilm and antifungal strategies has become increasingly urgent.

Major NAC pathogens

Among the NAC species, *C. auris* has emerged as a major global concern since its first isolation from a bloodstream infection in South Korea in 1996 [7]. It is now recognized as a healthcare-associated pathogen, causing hospital outbreaks and life-threatening infections worldwide [13]. Furthermore, it is difficult to identify, exhibits multidrug resistance, spreads rapidly in clinical settings, and persists on skin and environmental surfaces [7]. It is increasingly documented in bloodstream infections, with higher rates of reoccurrence and treatment options unfortunately becoming more convoluted due to the growing number of multidrug-resistant strains [13]. Six clades have been identified, with clades I, III and IV responsible for most of the outbreaks worldwide [7]. Mortality rates for *C. auris* candidemia are approximately 30%, though they vary by region, patient demographics and underlying conditions [7]. Resistance patterns differ among clades, but high levels of azole resistance are common [7], and individuals with prolonged hospitalizations, invasive procedures, or extensive antimicrobial exposure are at the greatest risk [7]. Despite targeted decolonization efforts, *C. auris* has proven exceptionally difficult to eradicate, leaving affected patients at an even higher risk for developing additional infections [13].

*C. glabrata* (*Nakaseomyces glabratus*) is another important opportunistic pathogen, typically residing in the gastrointestinal tract but capable of causing systemic disease in vulnerable patients [2]. It is now the second most common cause of candidemia in many regions and is increasingly recognized as a cause of vaginal candidiasis [2]. Its virulence is associated with immune evasion, adherence to host cells, dimorphism, phenotype switching, hydrolytic enzyme activity, and biofilm formation, all of which contribute to poor clinical outcomes [2]. Although reclassified taxonomically, the name *C. glabrata* is retained here for consistency with most of the published literature [2].

*C. parapsilosis*, which is a part of the psilosis complex together with *Candida orthopsilosis* and *Candida metapsilosis*, is frequently implicated in IC [1] and is particularly associated with hospital acquired infections. It is especially prevalent among low-birth weight neonates, immunocompromised patients, and individuals with prosthetic or implanted medical devices [1]. Its pathogenicity is largely driven by its ability to adhere to abiotic surfaces and to form biofilms on medical devices and host tissues [1]. In some regions, bloodstream infections caused by *C. parapsilosis* exceed those attributed to *C. albicans*, underscoring its growing clinical relevance [1].

Anandamide as a potential antifungal agent

Endocannabinoids are endogenous signaling molecules with diverse physiological functions including neuromodulatory, immunomodulatory, anti-inflammatory, and antioxidant activities [14]. Among them, N-arachidonoylethanolamine (anandamide; AEA), first identified by Mechoulam and colleagues in the 1990s [15], has more recently attracted attention for its antimicrobial properties against a range of bacterial pathogens [14,16,17]. Previously, our group demonstrated that AEA disrupts bacterial membrane integrity, resulting in membrane hyperpolarization, increased permeability, growth inhibition, altered membrane structure and rigidity, inhibition of drug efflux, and impaired glucose uptake into the cells of *Streptococcus mutans* and *Staphylococcus aureus* [16,18,19], but whether these membrane-targeting effects extend to *Candida* species remains unknown.

Additionally, a previous study on *Candida* demonstrated that AEA inhibits the yeast–hyphae transition, hyphal extension, and hyphal adherence to epithelial cells [20]. However, these findings are limited to a single *C. albicans* strain [20], leaving the activity of AEA against other clinically important NAC species largely unexplored.

Cytotoxicity levels were determined at 100 µg/mL AEA on epithelial cells [16]; however, AEA has been associated with both beneficial and adverse biological effects depending on the experimental model, receptor engagement, and tissue type [21]. This present work represents an in vitro mechanistic study intended to establish antifungal activity rather than therapeutic safety.

Given the urgent need for new therapeutic strategies to combat NAC infections, particularly those involving biofilms and drug-resistant strains, and our previous findings on AEA’s antimicrobial activities, this study investigates the antifungal activity of AEA against the three pathogenic NAC species described above. We aim to study species-specific differences in their susceptibility following AEA exposure, analyze how the lipid membrane may play a role in AEA’s action on *Candida*, as well as investigate why there are species-specific differences in their susceptibility to AEA. We hypothesized that AEA exhibits antifungal activity against NAC species by disrupting cellular membrane integrity and inducing downstream stress responses that impair fungal viability.

## 2. Materials and Methods

### 2.1. Stock Solutions

Anandamide (AEA) (>98.0% purity, Cayman Chemical, Ann Arbor, MI, USA) was dissolved in absolute ethanol at a concentration of 50 mg/mL; fluconazole (B. Braun, Barcelona, Spain) was prepared as a 2 mg/mL stock solution in DMSO. The compounds were diluted in RPMI 1640 medium to the desired working concentrations. Ethanol (0.025%, *v*/*v*), fluconazole and untreated *Candida* cells were used as experimental controls in all experiments unless noted otherwise.

### 2.2. Yeast Strains and Growth Conditions

Strains used: *Candida albicans* ATCC 90028, *Candida glabrata* ATCC 15126, *Candida parapsilosis* ATCC 22019, *Candidozyma auris* ATCC MYA-5001 and *Candidozyma auris* ATCC MYA-5002. Additionally, five clinical isolates of *C. auris* were provided from a collection of the Clinical Mycology Laboratory, Hadassah Medical Center, Israel: B12896, B11247, B12322, 7115A-1 and 3089A-2. Strains were cultured on Sabouraud dextrose agar (SDA, HiMedia Laboratories, Mumbai, India) at 35 °C for 24–48 h [22].

For planktonic growth, colonies were taken from SDA plates and suspended in RPMI-1640 medium (Sigma Aldrich, St. Louis, MO, USA) supplemented with L-glutamine, and buffered with 4-Morpholinopropanesulfonic Acid (MOPS; Formedium Ltd., Swaffham, UK) to pH 7.0; cell density was adjusted to a McFarland standard of 0.5 (equivalent to 0.15 OD_600nm_). That correlates to 1 × 10^6^ CFU/mL for *C. auris*, 7 × 10^7^ CFU/mL for *C. glabrata*, and 1.0 × 10^6^ CFU/mL for *C. parapsilosis*.

For biofilm formation assays, colonies were inoculated into yeast extract peptone dextrose (YPD) medium (4 g yeast extract, 8 g proteose peptone (BD Difco, BD Biosciences, Sparks, MD, USA), 8 g dextrose in distilled water to a final volume of 400 mL). Aliquots of 30 mL YPD in 250 mL Erlenmeyer flasks were inoculated with freshly grown colonies and incubated overnight at 30 °C with shaking at 150 rpm under aerobic conditions. All phenotypic assays were performed using cells harvested from the same standardized 24 h culture, washed twice with sterile PBS and resuspended in RPMI-1640 (L-glutamine, MOPS-buffered), also resuspended to OD_600_ = 0.15–0.2 prior to experimentation [22].

Biofilms were established by seeding 100 µL of standardized yeast suspension into each well of a tissue culture-treated flat bottom 96-well microtiter plate (Corning, Glendale, AZ, USA) in the absence or presence of increasing concentrations of AEA (0–250 µg/mL). Plates were incubated at 35 °C in aerobic conditions for 24 h [22]. Cell preparation was performed as described in Section 2.1 before all phenotypic assays. Untreated cells, ethanol (0.025%), and fluconazole-treated cells served as controls for all experiments unless noted otherwise.

### 2.3. Antifungal Susceptibility Testing

Antifungal susceptibility testing was performed using a broth microdilution assay modified from the Clinical and Laboratory Standards Institute (CLSI) reference method [23]. Briefly, yeast was cultured on SDA plates at 35 °C for 24–48 h. After this, colonies were taken from SDA plates and suspended in PBS medium and adjusted to McFarland standard 0.5. Then, cells were resuspended in RPMI-1640 medium buffered with MOPS at pH 7.0 to 1:1000 dilution, seeded in sterile transparent U-bottom 96-well microplates (Sterilin^TM^, Thermo Fisher Scientific, Loughborough, UK) and incubated in the presence or absence of AEA for 24 h at 35 °C. After incubation, growth was visualized in a microscope to determine the MIC and MBIC values.

### 2.4. Growth Curve Analysis

Planktonic yeast cultures were prepared as described in Section 2.2. Suspensions of 200 µL of *Candida* were seeded into tissue culture-treated flat bottom 96-well microtiter plates (Corning, Glendale, AZ, USA), in the absence or presence of increasing concentrations of AEA (0–125 µg/mL). Plates were incubated at 35 °C in a Multiskan SkyHigh microplate reader (Thermo Scientific, Life Technologies Holdings Pte Ltd., Singapore) for 24 h, and OD_540nm_ was recorded every 30 min [16]. Reductions in values were calculated using the following equation:

((Value of Control − Value of Sample)/(Value of Control)) ×100 = % reduction.(1)

### 2.5. Intracellular ATP Levels

The BacTiter-Glo™ microbial viability kit (Promega, Madison, WI, USA) was used to quantify the ATP levels in AEA- treated and untreated cells, according to the manufacturer’s instructions. Briefly, biofilms were prepared as described above by incubating yeast cultures with increasing concentrations of AEA (0–125 µg/mL) at 35 °C for 24 h. Following incubation, 50 µL of biofilm suspension was mixed with 50 µL of BacTiter-Glo reagent in a 96-white μ-Clear white 96-well plate (Greiner Bio-One, Frickenhausen, Germany), and the luminescence was recorded after 5 min using the Tecan Infinite M200 PRO plate reader (Tecan Trading AG, Männedorf, Switzerland) [16].

### 2.6. Metabolic Activity Assay

Metabolic activity of *Candida* biofilms was assessed using the 3-(4,5-Dimethyl-2-thiazolyl)-2,5-diphenyl-2H-tetrazolium bromide (MTT) assay (GoldBio, St. Louis, MO, USA), with modifications from previously described protocols [16]. Briefly, biofilms were prepared as described above in the presence or absence of AEA (0–125 µg/mL) and incubated at 35 °C under aerobic conditions [22]. After 24 h, MTT solution was added to each well to a final concentration of 0.5 mg/mL, and plates were incubated for an additional 4 h at 35 °C. Formazan production was quantified by measuring absorbance at 570 nm using a Tecan infinite M200 PRO plate reader.

### 2.7. Biofilm Analysis by Spinning Disk Confocal Microscopy (SDCM)

Spinning disk confocal microscopy (SDCM) was used to examine biofilm structure and assess live/dead cell distribution following AEA treatment. Biofilms were formed as described above and 200 µL standardized cell suspension were seeded into µ-Slide 8 Well ibiTreat chamber slides (ibidi GmbH, Gräfelfing, Germany) for 24 h at 35 °C. After incubation, biofilms were gently washed twice with sterile PBS. Protocol for fluorescence staining and analysis was adapted from Kagan et al. [22].

#### 2.7.1. Fluorescence Staining

Live/dead staining was performed as previously described [22]. Biofilms were incubated with 3.3 µM SYTO 9 (Molecular Probes, Life Technologies, Carlsbad, CA, USA), and 10 µg/mL of propidium iodide (PI; Sigma, St. Louis, MO, USA) for 30 min at room temperature. Following staining, biofilms were gently washed with PBS and fixed with 4% paraformaldehyde in PBS for 20 min at room temperature. After fixation, samples were washed again with PBS and mounted in 50% glycerol.

#### 2.7.2. Confocal Microscopy and Fluorescence Quantification

Confocal microscopy was performed with a Nikon Yokogawa W1 Spinning Disk Confocal Microscope (Nikon Corporation, Tokyo, Japan) [16]. SYTO 9 fluorescence (green), which labels both live and dead cells, was visualized using 488 nm excitation and 515 nm emission filters. PI fluorescence (red), which penetrates only cells with perforated membranes, was detected using 543 nm excitation and 570 nm emission filters. Images were analyzed using the NIS-Element AR software (version 6.10.00). Quantitative analysis was performed using QuPath (version 0.5.1), where SYTO 9-positive and PI-positive nuclei were segmented using QuPath’s pixel thresholding tool. The ratio of PI-positive area to SYTO 9-positive area was calculated as a measure of cell death. Additional image analysis was performed using CellProfiler (version 4.2.8, Broad Institute Cambridge, MA, USA) to analyze changes in growth parameters and staining intensity in the cells.

### 2.8. Membrane Permeability Assay

Membrane potential changes were assessed using the fluorescent probe DiS-C3(3) (3,3- dipropylthiacarbocyanine iodide; Sigma-Aldrich, St. Louis, MO, USA). Biofilms were prepared as described above in Section 2.2. An amount of 1 mL of standardized cell suspension was incubated in 1.7 mL clear microtubes (Corning, Glendale, AZ, USA) for 24 h at 35 °C. Following incubation, all samples were adjusted to an OD_600nm_ of 0.2, washed twice with PBS, and resuspended in 1 mL of room temperature 10 µM DiS-C3(3) (diluted in PBS) where they were then read immediately. An unstained sample served as a negative control. Aliquots of 200 µL from each sample were transferred into black µClear 96-well plates (Greiner Bio-One, Frickenhausen, Germany). Fluorescence was measured using a Tecan Infinite M200 PRO plate reader (Tecan Trading AG) with excitation at 531 nm and emission recorded from 560 to 590 nm. Readings were taken every 5 min for 120 min, with gentle shaking before each measurement. Membrane depolarization was quantified by calculating the fluorescence ratio:

R(t) = I580(t)/I560(t)(2)

where R(t) is the fluorescence intensity ratio at time *t*, I580(t) is the fluorescence emission intensity measured at 580 nm at time *t*, and I560(t) is the fluorescence emission intensity measured at 560 nm at time *t* [24].

### 2.9. Membrane Fluidity Assay

The membrane fluidity assay was adapted from previously described protocols with modifications [25,26]. Biofilms were prepared as described in Section 2.8. Samples were resuspended in PBS and Laurdan (AnaSpec, Fremont, CA, USA) was added to each sample at a final concentration of 5 µM; cells were incubated for 30 min at 35 °C in the dark. An unstained sample served as control. Following incubation, cells were centrifuged, washed twice, and resuspended in PBS. Aliquots of 200 µL were transferred into black µClear black 96-well plates (Greiner Bio-One, Frickenhausen, Germany). Fluorescence measurements were performed using the Tecan Infinite M200 PRO plate reader (Tecan Trading AG) with excitation at 366 nm and emission recorded at 400–550 nm. Membrane fluidity was quantified by calculating the General Polarization (GP) value using the following equation:

GP = (I440 − I490)/(I440 +I490),(3)

where I440 and I490 are the fluorescence intensities at 440 and 490 nm, respectively. A higher GP value denotes a more rigid membrane while a lower GP value suggests a more fluid one [26].

### 2.10. ROS Production

ROS production by NAC cells was measured using the fluorometric assay 2′,7′-dichlorofluorescein diacetate (DCFH-DA) (Sigma-Aldrich, St Louis, MO, USA) as described in [27]. Biofilms were prepared as described above in Section 2.8. Samples were resuspended in PBS and DCFH-DA was added to a final concentration of 10 µM in PBS and incubated at 35 °C for 30 min in the dark. An unstained sample served as control. After incubation the cells were washed twice and resuspended in PBS. Aliquots of 200 µL were transferred into black µClear 96-well plates (Greiner Bio-One, Frickenhausen, Germany). The excitation was measured at 485 nm and emission at 540 nm in a Tecan Infinite M200 PRO plate reader (Tecan Trading AG).

### 2.11. Efflux Assay

The efflux assay was adapted from Chamlagain et al. [28] with slight modifications. Overnight YPD NAC cultures were washed twice with glucose-free PBS and resuspended in 10 µM Rhodamine 6G (R6G) (Sigma Aldrich, St. Louis, MO, USA) and thereafter resuspended in glucose-free PBS to 0.5 OD_600nm_. Cells were incubated for 30 min at 35 °C, then washed five times with glucose-free PBS to remove extracellular R6G. Then, samples were resuspended in PBS with 2% glucose, treated with 62.5 and 125 µg/mL AEA, and incubated at 35 °C. At 0, 15, 30, 60, 90 and 120 min; 300 µL aliquots were collected, centrifuged (12 rpm, 2 min), and the supernatant transferred to wells of a black µClear 96-well plate (Greiner Bio-One, Frickenhausen, Germany). Fluorescence was measured at 529 nm excitation and 553 nm emission using a Tecan Infinite M200 PRO plate reader (Tecan Trading AG). An unstained sample served as a control.

### 2.12. Statistical Analysis

All experiments were performed using three independent biological replicates. Where applicable, measurements were performed in technical triplicates. Data are presented as a mean ± standard deviation. Statistical analyses were performed using Student’s *t*-test in Microsoft Excel for Microsoft 365 (Version 2108, Microsoft Corp., Redmond, WA, USA). Comparisons between groups were performed using a two-tailed unpaired Student’s *t*-test, with a *p*-value of less than 0.05 considered significant when comparing treated versus control samples.

## 3. Results

### 3.1. Anandamide Attenuates Growth of Candida Strains

The minimum inhibitory concentration (MIC) and minimum biofilm inhibitory concentration (MBIC) of AEA for different *Candida* strains were determined based off the CLSI protocol with slight modifications, and values are presented in Table 1 and Table 2.

**Figure 1:**
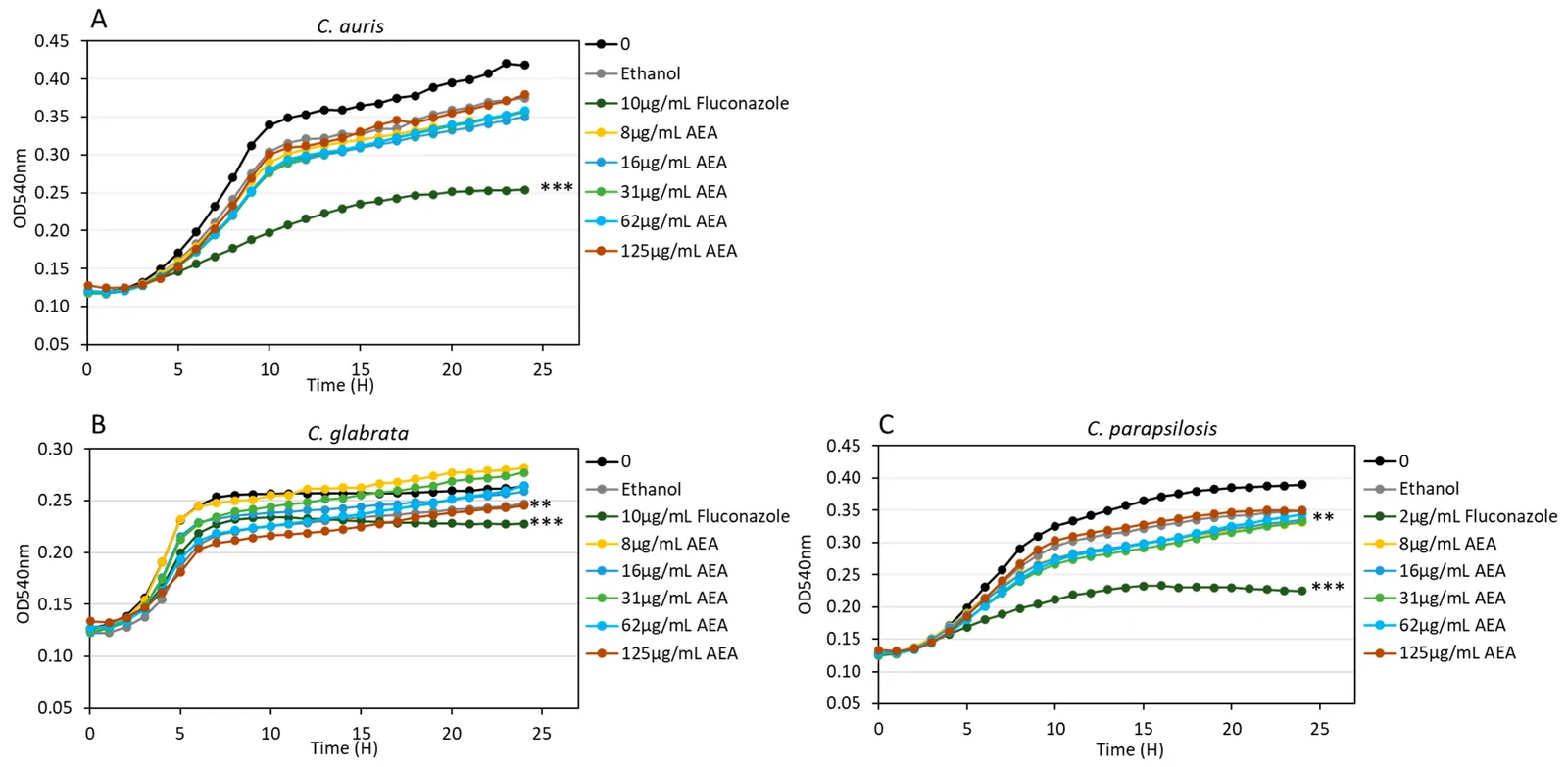
AEA attenuated the growth of NAC strains. Kinetic growth curves of *C. auris* (**A**), *C. glabrata* (**B**), and *C. parapsilosis* (**C**) incubated with increasing concentrations of AEA (0–125 µg/mL) as measured by OD_540nm_. Ethanol, fluconazole and untreated cells were used as controls. *n* = 3; ** *p* < 0.01, *** *p* < 0.001 in comparison with untreated controls.

The following AEA-responding strains were selected for subsequent experiments: *C. glabrata* ATCC 15126, *C. parapsilosis* ATCC 22019, and *C. auris* MYA-5001. Growth kinetics were assessed by monitoring the optical density (OD) of planktonic cultures of *C. auris* (Figure 1A), *C. glabrata* (Figure 1B), and *C. parapsilosis* (Figure 1C), incubated with increasing concentrations of AEA (8–125 µg/mL) for 24 h. Dose-dependent attenuation of fungal growth was observed for all three NAC strains (Figure 1). Notably, *C. albicans* displayed markedly lower susceptibility to AEA in comparison to the NAC species examined, suggesting species-specific differences in sensitivity to this compound.

### 3.2. The Impact of AEA on Biofilm Viability Determined by MTT Metabolic Assay and Intracellular ATP Levels

**Figure 2:**
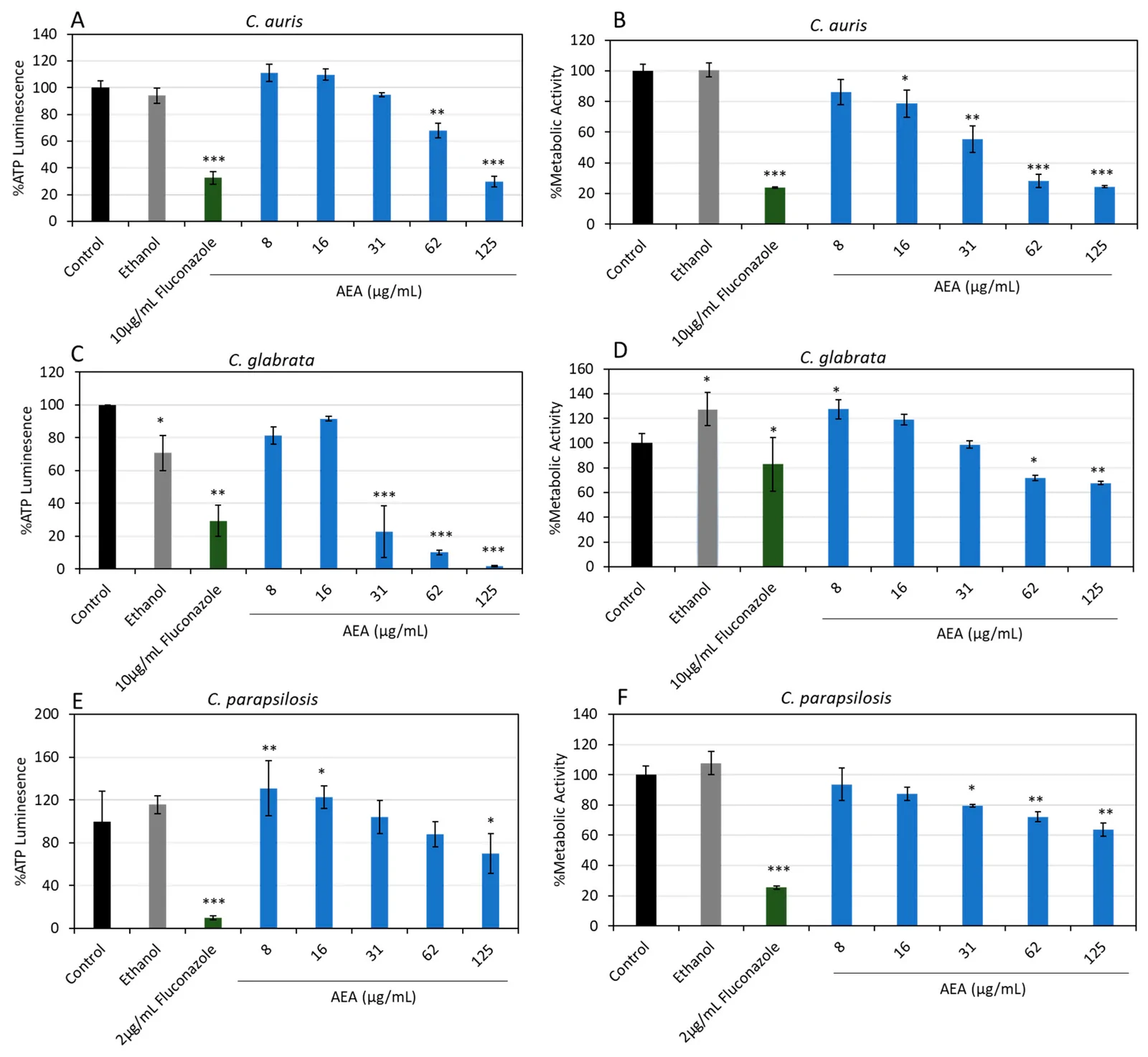
AEA causes dose-dependent reductions in ATP production and metabolic activity. *C. auris* (**A**,**B**), *C. glabrata* (**C**,**D**), and *C. parapsilosis* (**E**,**F**) were treated with increasing concentrations of AEA (0–125 µg/mL) for 24 h and subsequently assessed for ATP production and metabolic activity (MTT assay). Ethanol, fluconazole and untreated cells were used as controls. *n* = 3; * *p* < 0.05, ** *p* < 0.01, *** *p* < 0.001 in comparison with untreated controls.

Across all three NAC species, AEA induced a clear dose-dependent reduction in both metabolic activity and intracellular ATP levels compared with untreated controls, with significant decreases observed at 125 µg/mL (Figure 2; *p* < 0.05). The greatest inhibitory effects were detected in *C. auris* (Figure 2A,B) and *C. glabrata* (Figure 2C,D) in contrast to *C. parapsilosis*. At 125 µg/mL AEA, *C. auris* exhibited >70% reduction in ATP levels (*p <* 0.001) and in metabolic activity (*p* < 0.001), while *C. glabrata* showed a 98 ± 3% decrease in ATP levels (*p* < 0.001) and a 32 ± 2% decrease in metabolic activity (*p* < 0.01). *C. parapsilosis* also demonstrated inhibition, though to a lesser extent, with 30 ± 2% and 36 ± 4% reductions in ATP production and metabolic activity (*p* < 0.05) in (*p* < 0.01) at 125 µg/mL AEA.

### 3.3. AEA Increases Membrane Fluidity

**Figure 3:**
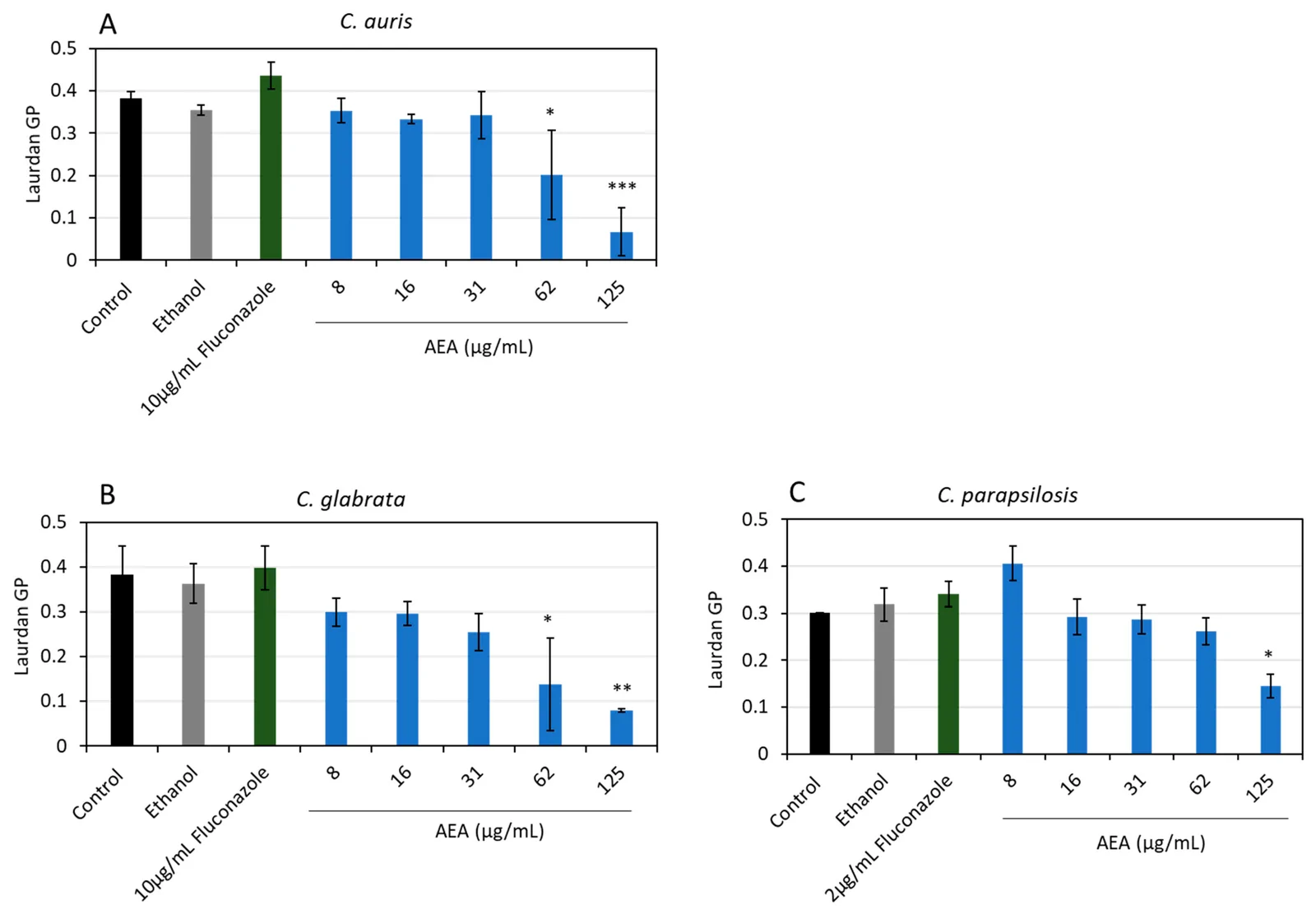
AEA increases membrane fluidity. *C. auris* (**A**), *C. glabrata* (**B**), and *C. parapsilosis* (**C**) treated with varying concentrations of AEA (8–125 µg/mL) for 24 h were stained with Laurdan and assessed for changes in membrane fluidity. Fluorescence was measured using an excitation wavelength of 366 nm and an emission range of 400–550 nm. Ethanol, fluconazole and untreated cells were used as controls. *n* = 3; * *p* < 0.05, ** *p* < 0.01, *** *p* < 0.001 in comparison with untreated controls.

Membrane fluidity was assessed using Laurdan-based General Polarization (GP) measurements following 24 h exposure to AEA [29]. No significant changes in GP values were observed at 8–31 µg/mL in any of the *Candida* species tested in comparison with untreated controls; however, higher AEA concentrations resulted in marked decreases in GP values for all three NAC strains, indicating increased membrane fluidity (Figure 3). In comparing at 125 µg/mL AEA, the greatest effect was seen in *C. auris*, where GP values decreased by 82 ± 6% (Figure 3A; *p* < 0.001), in comparison to *C. glabrata*, which exhibited GP reductions of 79 ± 2% (Figure 3B; *p* < 0.01), and finally *C. parapsilosis*, which showed a 52 ± 0% decrease (Figure 3C; *p* < 0.05).

### 3.4. AEA Induces Depolarization of the Membrane

**Figure 4:**
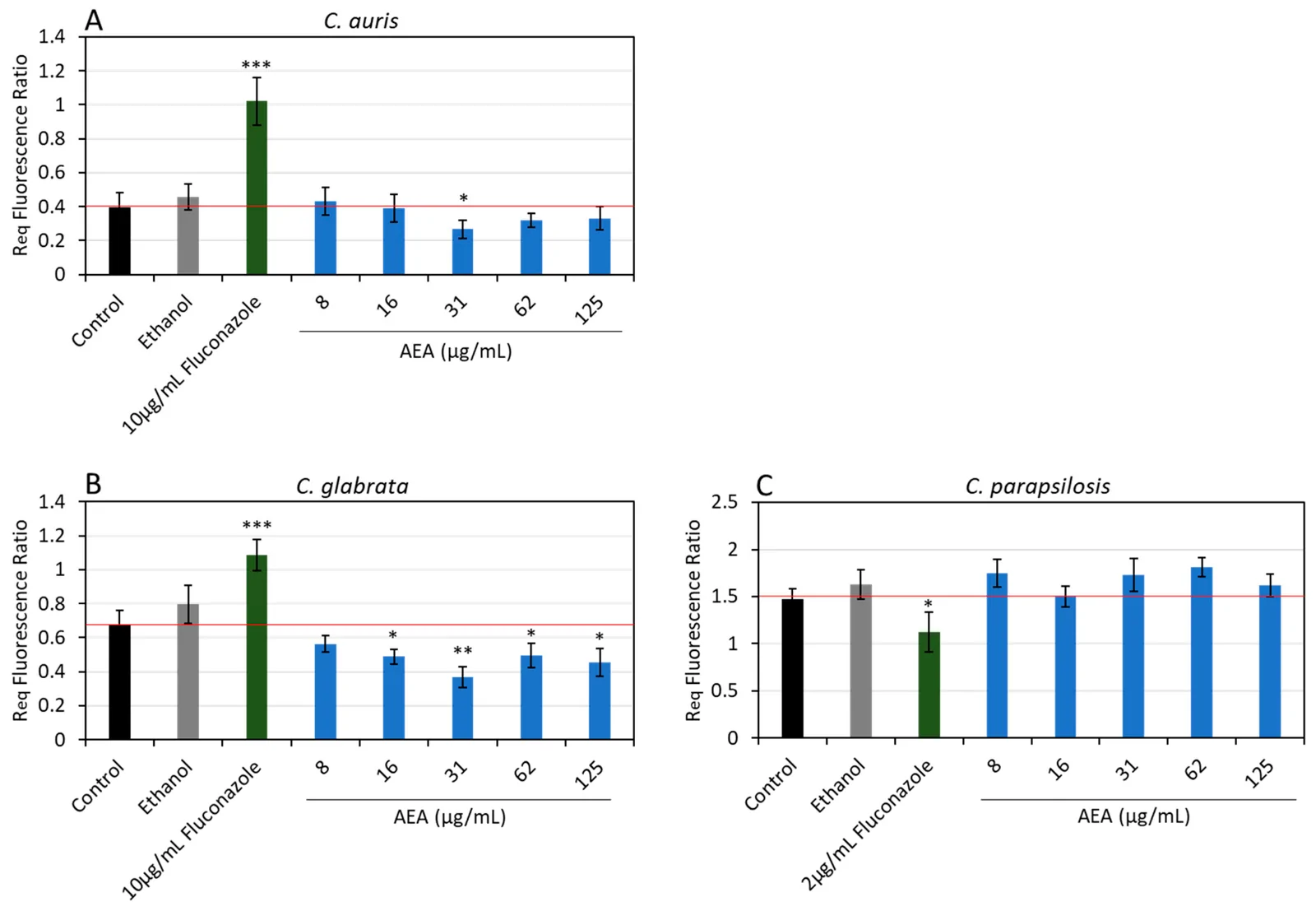
Membrane polarization of *Candida* after AEA treatment. *C. auris* (**A**), *C. glabrata* (**B**), and *C. parapsilosis* (**C**) treated with varying concentrations of AEA (8–125 µg/mL) for 24 h and stained with DiS-C3(3) were assessed for polarization of the membrane. The fluorescence of the ratio equilibrium (Req) (**A**–**C**) was assessed using a fluorescence intensity scan from 560 to 590 nm every 4 min for 120 min. Ethanol, fluconazole and untreated cells were used as controls. Red line depicts control value. *n* = 3; * *p* < 0.05, ** *p* < 0.01, *** *p* < 0.001 in comparison with untreated controls.

Membrane potential changes were assessed using the potentiometric dye DiS-C3(3) following 24 h exposure to AEA. AEA treatment resulted in modest membrane depolarization in *C. glabrata* (Figure 4B; *p* < 0.05). In contrast, only minimal depolarizing effects were observed in *C. auris* (Figure 4A; not significant) and slight hyperpolarization in *C. parapsilosis* (Figure 4C; not significant). As a reference control, fluconazole induced hyperpolarization in *C. auris* (*p* < 0.001) and *C. glabrata* (*p* < 0.001), while causing depolarization in *C. parapsilosis* (*p* < 0.05). These findings indicate that AEA affects membrane potential in *C. glabrata* to a greater extent, with comparatively limited effects on the other species.

### 3.5. Inhibition of Efflux Pump Activity by AEA

To determine whether AEA exerts rapid effects on *Candida* membrane function, real-time efflux assays were performed using the fluorescent substrate Rhodamine 6G (R6G), a reporter of ABC-type efflux pump activity in *Candida*. *C. auris* and *C. glabrata* cells were stained with R6G and subsequently exposed to 62.5 or 125 µg/mL AEA. Efflux was initiated by adding glucose, and extracellular R6G fluorescence was monitored over time.

**Figure 5:**
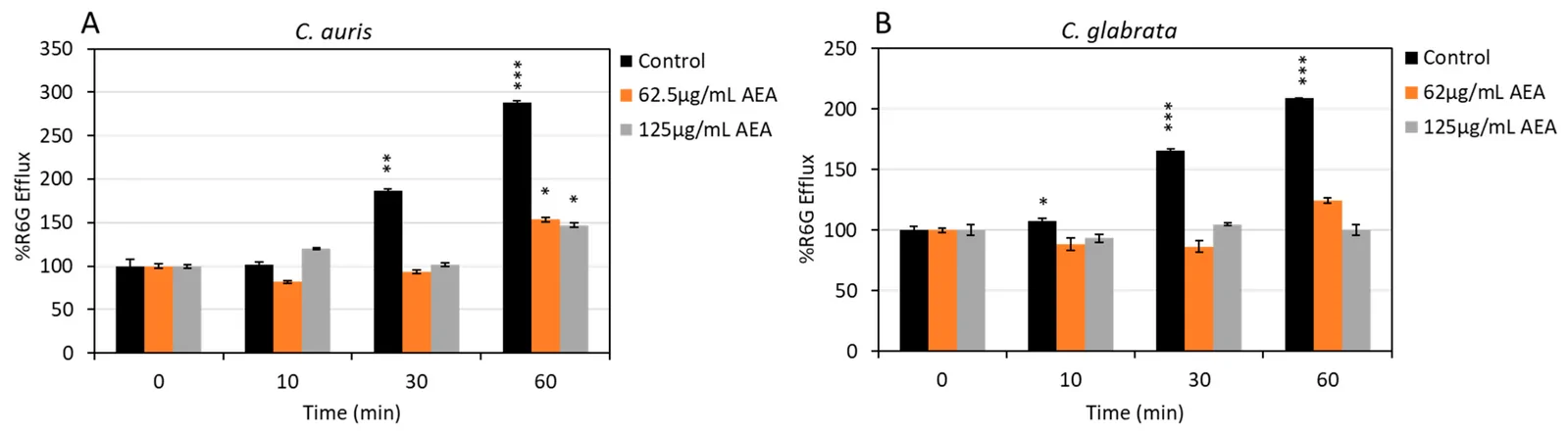
Immediate effect on efflux pumps activity after AEA treatment. (**A**) *C. auris* and (**B**) *C. glabrata* cells were loaded with Rhodamine 6G (R6G) and subsequently treated with AEA at concentrations of 62.5 and 125 µg/mL. Fluorescence of extracellular R6G was measured using an excitation of 485 nm, and emission of 525 nm. Untreated cells and unstained cells were used as controls. *n* = 3; * *p* < 0.05, ** *p* < 0.01, *** *p* < 0.001 compared with the untreated control.

In untreated control samples, both *C. auris* (Figure 5A) and *C. glabrata* (Figure 5B) displayed a clear and progressive increase in extracellular R6G fluorescence (*p* < 0.001), consistent with active efflux pump function. In contrast, AEA-treated cells exhibited markedly reduced R6G release at both concentrations tested, indicating rapid inhibition of efflux activity. This suppression of R6G extrusion suggests that AEA rapidly interferes with membrane-associated transport processes, consistent with the membrane-targeting effects observed in the 24 h assays.

Together, these findings suggest that AEA rapidly impairs efflux pump function within minutes of exposure, providing mechanistic support for its ability to sensitize *Candida* cells and impair biofilm activity.

### 3.6. AEA Induces Immediate Reactive Oxygen Species (ROS) Production

**Figure 6:**
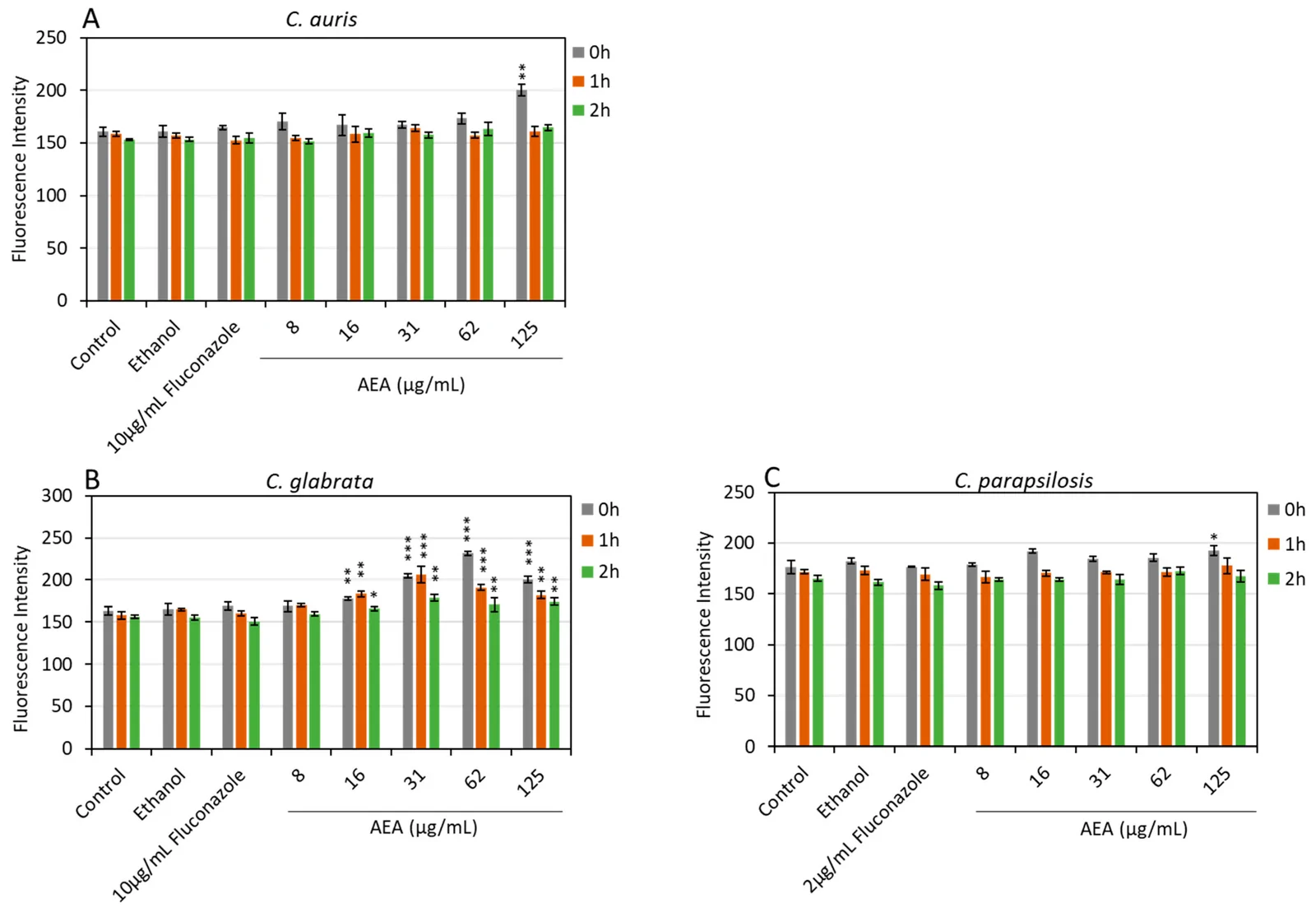
Immediate ROS production after AEA treatment. *C. auris* (**A**), *C. glabrata* (**B**), and *C. parapsilosis* (**C**) were treated with increasing concentrations of AEA (8–125 µg/mL) for 0, 1 and 2 h and assessed for ROS production. Fluorescence was assessed using an excitation wavelength of 366 nm, and an emission range of 400–550 nm. Fluconazole and ethanol treated cells were used as controls. *n* = 3; * *p* < 0.05, ** *p* < 0.01, *** *p* < 0.001 compared with untreated controls.

ROS generation was assessed using the DCFH-DA fluorescent ROS probe following exposure to AEA. Rapid increases in ROS levels were observed in all three NAC species, with the strongest and longest lasting effect observed in *C. glabrata* (Figure 6B). *C. glabrata* exhibited sustained ROS production that gradually declined over 2 h (*p* < 0.01), in contrast to *C. auris*, where it returned after 1 h (Figure 6A; *p* < 0.01), and *C. parapsilosis*, which had even a weak increase in ROS after immediate treatment and only at the highest concentration of AEA (Figure 6C; *p* < 0.05). These findings indicate that AEA induces a rapid, concentration-dependent ROS response in *C. glabrata*; a weaker production of ROS was observed only after immediate treatment in *C. auris* and *C. parapsilosis*.

### 3.7. Live/Dead Staining of AEA-Treated Candida Biofilms Using Spinning Disk Confocal Microscopy (SDCM)

To further evaluate the impact of AEA on biofilm cell viability, NAC biofilms were stained with SYTO 9 and PI. Based on their higher sensitivity to AEA in earlier assays, *C. auris* and *C. glabrata* were selected for SDCM analysis.

**Figure 7:**
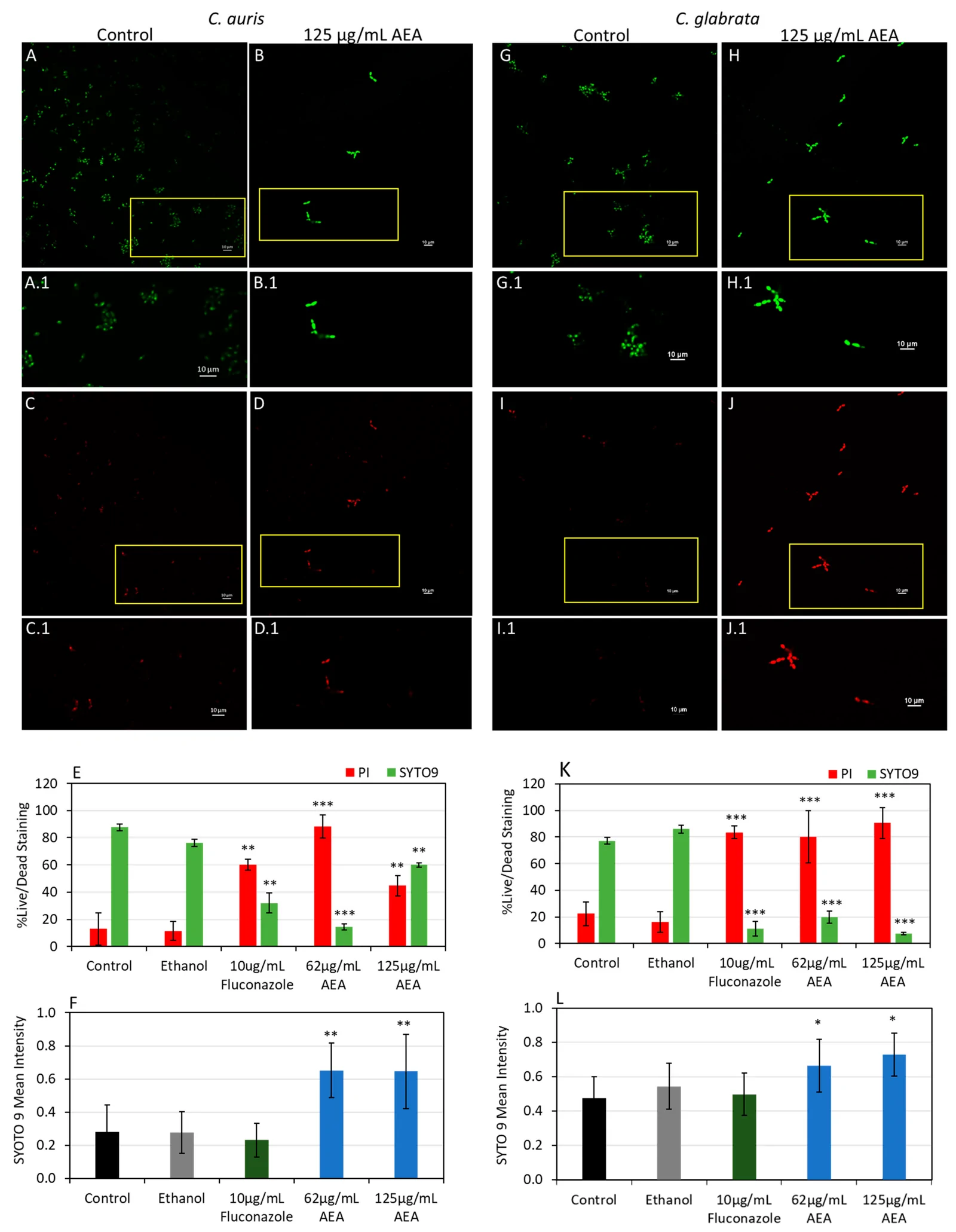
SYTO 9/PI staining of NAC biofilms reveal increased membrane permeability following AEA treatment. *C. auris* (**A**–**D**) and *C. glabrata* (**G**–**J**) were treated with 62.5 or 125 µg/mL AEA for 24 h and subsequently stained with SYTO 9 and PI for live/dead cell assessment. Fluconazole-treated and ethanol-treated cells served as controls. Yellow frames indicate sections that have been zoomed in on for better visualization (**A.1**–**D.1**) and (**G.1**–**J.1**). The SYTO 9/PI ratio was quantified using Nikon NIS Element software (**E**,**K**), and fluorescence intensity was analyzed using CellProfiler (V4.2.8) (**F**,**L**). Data represents analysis of six images per condition, corresponding to approximately 50–2500 individual cells. Scale bar = 10 µm; * *p* < 0.05, ** *p* < 0.01, *** *p* < 0.001 compared with untreated controls.

Biofilms of *C. auris* (Figure 7A,B) and *C. glabrata* (Figure 7G,H) were treated with 125 µg/mL of AEA for 24 h and subsequently analyzed by SDCM. Image analysis revealed a marked reduction in the total number of cells in AEA-treated *C. auris* (Figure 7B) and *C. glabrata* (Figure 7H) compared with untreated controls (Figure 7A,G). Quantification of the SYTO 9/PI ratio demonstrated a substantial increase in PI-positive cells following AEA exposure (Figure 7D,J). In *C. auris*, PI-positive cells increased from an average of 12 ± 9% in untreated biofilms to 88.2% following treatment with 62.5 µg/mL AEA (Figure 7E; *p* < 0.001). Similarly, *C. glabrata* showed an increase from 22 ± 3% in untreated samples to 80 ± 0% with 62.5 µg/mL AEA (Figure 7K; *p* < 0.001), with elevated PI staining maintained at 125 µg/mL AEA (Figure 7J,K; *p* < 0.001).

However, *C. auris* biofilms treated with 125 µg/mL AEA displayed a higher proportion of SYTO 9-positive cells (55 ± 4%), relative to PI-positive cells (44 ± 6%), (Figure 7E *p* < 0.01), despite a marked reduction in total cell numbers (Figure 7B). This discrepancy suggested altered dye retention rather than increased viability. To further investigate this phenomenon, SYTO 9 fluorescence intensity was quantified. AEA-treated cells exhibited a significant increase in SYTO 9 retention (Figure 7B.1,H.1) compared with untreated controls (Figure 7A.1,G.1) in both species, with the effect particularly pronounced in *C. auris* (Figure 7F; *p* < 0.01 and Figure 7L *p* < 0.05). These results indicate that AEA-treatment enhances SYTO 9 retention, likely due to altered membrane permeability or impaired dye efflux, and that SYTO 9 positivity under these conditions reflects cell count, both live and dead, while not necessarily reflecting cell viability.

## 4. Discussion

Previously, our group studied the effects of AEA against *Streptococcus mutans* and demonstrated that AEA exhibits antibacterial and antibiofilm activities. Specifically, AEA targeted the bacterial membrane, leading to membrane depolarization, increased permeability, growth inhibition, and reduced glucose uptake by the cells [16,18]. When AEA was tested on *C. albicans,* it was found to inhibit the adherence of the *Candida* cells onto cervical epithelial cells and repressed filamentous growth [20]. These findings prompted us to investigate the antifungal potential of AEA against clinically relevant NAC species. Although *Candida* species share similar major phospholipid classes in their membranes, studies assessing membrane compositions have shown that membrane lipids differ significantly in their fatty acyl chains and sphingolipid content [30]. As a lipid-derived compound, AEA is expected to interact differently with distinct fungal membranes, potentially resulting in species-specific effects, as observed in the present study.

The minimum inhibitory concentration (MIC) required to inhibit growth was determined for *C. albicans*, *C. glabrata*, and *C. parapsilosis* (Table 1), as well as for *C. auris* strains representing various clades (Table 2). *C. albicans* ATCC 90028 exhibited no detectable susceptibility to AEA at concentrations up to 500 µg/mL, in contrast to *C. glabrata* and *C. parapsilosis* (Table 1). Among the *C. auris* strains tested, MYA-5001 displayed the greatest susceptibility to AEA. Notably, MYA-5001 belongs to clade II, which has been reported to be more susceptible to fluconazole than clades I, III, and IV [31,32]. The latter clades are more likely to harbor mutations in the *Erg11* gene, which encodes the azole target enzyme as well as additional mutations associated with increased antifungal resistance [31,32]. In contrast, such mutations have not been detected in clade II strains [31]. Based on their heightened susceptibility to AEA, the following strains were selected for further study: *C. auris* MYA-5001, *C. glabrata* ATCC 15126, and *C. parapsilosis* ATCC 22019.

AEA-treated samples exhibited a reduced growth rate compared to their respective untreated controls; however, the duration of the logarithmic phase remained largely unchanged between untreated and treated cells (Figure 1). A similar pattern was previously observed in *C. auris* treated with Withania somnifera seed oil (WSSO), which demonstrated reduced growth rates without altering the time required to reach the stationary phase [6]. Notably, with the exception of the MIC and MBIC assays (Table 1 and Table 2), all experiments were conducted using substantially higher OD. In this manner, AEA is observed to be density-dependent, requiring higher concentrations to affect the *Candida* cells, a feature also observed with the phytocannabinoid Cannabigerol [33]. These findings suggest that AEA acts in a dose-dependent manner and that it exerts a fungistatic rather than a fungicidal effect on *Candida* cells, similar to the mode of action commonly associated with azoles [34] and as observed in the present study (Figure 1).

A major mechanism responsible for high-level azole resistance and multidrug resistance (MDR) in clinical *Candida* isolates involves the overexpression of efflux pumps [34]. These pumps belong primarily to two families: ATP-binding cassettes (ABC) transporters and the major facilitator superfamily (MFS) transporters [34]. MFS transporters mediate the transport of a limited range of small solutes in response to chemiosmotic ion gradients, whereas ABC transporters exhibit a broader substrate specificity and rely on ATP hydrolysis for transport [34]. Given the central role of ABC transporters in MDR, targeting the ATP required for efflux pump activity has emerged as a potential therapeutic strategy [34] and would also disrupt the cells’ physiology, metabolic activity, and virulence [35]. The reduced ATP may reflect early metabolic dysfunction with downstream effects on both resistance mechanisms and pathogenicity [32,36]. AEA-treated cells showed reduced ATP levels and decreased metabolic activity after 24 h of exposure (Figure 2). Additionally, reduced ATP levels may contribute to the reduced efflux activity observed following AEA treatment (Figure 5) and could cause the intracellular accumulation of toxic compounds, like AEA, inside the cells. To further investigate how ATP depletion might influence cellular viability and membrane-associated function, we examined several membrane-associated parameters, including membrane potential, fluidity, and permeability [32,36].

Membrane fluidity is a critical determinant of fungal cell physiology, influencing permeability, membrane protein function, cellular homeostasis, drug transport and ATPase activities [6,37]. Increased membrane fluidity is associated with reduced lipid packing and altered sterol organization [29,38]. Because membrane lipids are essential for the activity of multidrug transporters in *Candida* [37], increased fluidity may compromise membrane integrity and disrupt the function of membrane-associated proteins, thus increasing susceptibility of the *Candida* to drug treatment [29,38]. In a novel lipidomic study, drug-susceptible and resistant clinical isolates of *C. auris* were studied to identify how the lipid composition of the membranes varied [39]. Within *C. auris* alone, membrane composition varies among clades and strains [39], which may explain the difference in susceptibility to AEA observed in this study (Table 2). The variability in responses between strains can be attributed, at least in part, to differences in plasma membrane structure and composition, in addition to other contributing factors. In fact, species-specific susceptibility among NAC species is not unusual; it has also been reported in response to other antifungal agents, including Thai *Piper betle* leaf extract, and has been attributed to differences in membrane composition, efflux pump activity, and metabolic adaptability [40].

In the present study, Laurdan, a membrane phase-sensitive fluorescent probe, was used to assess spatial changes in lipid organization [41]. AEA treatment reduced GP values across all NAC species tested, and the magnitude of this effect followed the order *C. auris* > *C. glabrata* > *C. parapsilosis*, (Figure 3). This indicates increased membrane fluidity which corresponds to a more disordered membrane state associated with reduced ergosterol content and altered lipid packing [41].

These findings were unexpected, as *C. parapsilosis* typically exhibits higher drug susceptibility compared with other NAC species [42]. However, in this study the strain tested showed the lowest susceptibility to AEA relative to *C. glabrata* and *C. auris.* A possible explanation could be how the defense mechanisms of *Candida* species are altered when they are incorporated into biofilms. Biofilms of *C. parapsilosis* exhibit stronger resistance to antifungals and predominantly have a carbohydrate-based extracellular matrix (EM) layer that sequesters antifungals before they can reach the cell membrane, with this effect especially seen in agents that target the membrane [43,44,45,46,47,48], such as AEA. In comparison, *C. glabrata* and *C. auris* utilize a combined defense against antifungal agents which relies on both the EM protection as well as on efflux resistance [43]. This multilayered defense system may in fact be the reason that targeting the lipid composition of *C. auris* and *C. glabrata* will cause weakened membrane defenses, which in turn leave them more vulnerable to membrane-targeting drugs like AEA [31].

Additionally, AEA induced modest membrane polarization effects on the three NAC species. A slight depolarization was observed in *C. glabrata* and *C. auris* while membrane hyperpolarization was observed in *C. parapsilosis*, albeit minimally (Figure 4). Polarization of the membrane can be influenced by many factors including the concentration of the drug being used as well as the lipid content and organization in the membrane being targeted [26,49,50]. The depolarizing effects noted in *C. auris* and *C. glabrata* could be caused by increased membrane permeability, which was observed in these species (Figure 7), thus leading to some loss of membrane integrity which could retard the electrochemical gradient and result in depolarization effects [26,51]. On the other hand, *C. parapsilosis* showed only minimal hyperpolarization after AEA treatment, which could be the result of AEA causing enough changes in the membrane sterol composition that ion movements are affected, resulting in slight hyperpolarization [49]. *Candida* species are generally osmotolerant; however, interspecies differences in salt tolerance and ion homeostasis have been observed [52]. While *C. albicans* is relatively osmotolerant, *C. parapsilosis* exhibits high tolerance to elevated salt concentrations and can survive at low intracellular K+/Na+ ratio [52]. The differential effects observed between these NAC species, as well as the limited effects of AEA on membrane potential despite increased membrane fluidity and permeability, may therefore reflect the ability of *Candida* cells to maintain intracellular ion homeostasis under stress [52]. *Candida* species utilize multiple transport systems to regulate alkali metal cation uptake and efflux, thereby preserving intracellular potassium-to-sodium balance, although the activity of these systems differs between species [52]. In this context, the preservation of membrane potential in *C. auris* and *C. parapsilosis* may indicate more effective compensatory mechanisms, such as enhanced ion transport or membrane remodeling, that help maintain electrochemical gradients despite structural membrane perturbations [52]. In contrast, the sustained depolarization observed in *C. glabrata* may reflect a reduced capacity to restore ion homeostasis or a higher energetic burden associated with maintaining membrane integrity under stress. In any case, without more investigative lipid composition studies, it is difficult to understand the exact mechanism occurring.

Membrane permeability in AEA-treated biofilms revealed marked disruption of membrane integrity in both *C. auris* and *C. glabrata* after 24 h of treatment (Figure 7). The marked increase in PI-positive cells (Figure 7C,G) indicates loss of membrane integrity and barrier function. These results are consistent with the elevated membrane fluidity detected following AEA treatment (Figure 3) and support the conclusion that AEA perturbs lipid organization, leading to enhanced membrane permeability. Interestingly, while *C. glabrata* displayed a sustained increase in PI staining across concentrations (Figure 7G), *C. auris* biofilms treated with higher AEA concentrations exhibited an apparent increase in SYTO 9-positive cells despite a marked reduction in total cell number (Figure 7C). This suggests that SYTO 9 retention under these conditions reflects altered membrane permeability rather than preserved viability.

Indeed, increased SYTO 9 fluorescence intensity following AEA treatment in both species, particularly in *C. auris* (Figure 7D), indicates enhanced intracellular dye accumulation, likely due to increased membrane permeability and impaired efflux activity (Figure 5). Consistently, Smriti et al. [37] reported that increased membrane fluidity in *C. albicans* was associated with reduced activity of the ABC transporter Cdr1p, resulting in intracellular accumulation of substrates such as Rhodamine 123 and β-estradiol. *Candida* species typically overexpress ABC and MFS transporters to overcome antifungal treatments leading to treatment failures, and this overexpression is also typically associated with reduced intracellular dye accumulation, such as was observed with SYTO 9 in *C. auris* [53]. To further evaluate this, R6G efflux assays were performed. Within the first hour of treatment, AEA-treated cells showed reduced R6G efflux compared to untreated controls in both *C. auris* and *C. glabrata* (Figure 5). These results suggest that AEA causes impaired efflux activity by membrane alterations which lead to increased intracellular drug retention and represent a promising strategy for overcoming multidrug resistance.

Targeting efflux pumps is becoming an increasingly popular area of research in NAC species. El-Ganiny et al. [54] demonstrated that Pantoprazole and haloperidole treated against various NAC species were able to chemosensitize fluconazole-resistant isolates by counteracting overexpressed CDR1, MDR1, and ABC2 efflux genes, resulting in increased intracellular drug accumulation [54]. Despite many recent studies utilizing and repurposing drugs as efflux pump inhibitors [55,56,57,58], the clinical translation of this remains limited. Unlike azoles, which primarily target *Erg11* and are susceptible to resistance arising from mutations or altered expression of a single gene [59,60], AEA’s disruption of the membrane integrity and associated membrane functions may be considered less vulnerable to resistance mediated by single-gene mutations because they affect multiple interconnected cellular processes simultaneously.

Increased susceptibility of *Candida* strains has been noted previously [24]. In the case of membrane fluidity on the ABC transporter Cdr1p in *C. albicans*, increased membrane fluidity significantly impaired substrate efflux activity [37]. This effect was associated with enhanced antifungal susceptibility and intracellular accumulation of fluorescent substrates inside the cell that are normally extruded by the pump, ultimately reducing the ability of Cdr1p to confer multidrug resistance [37]. In addition, *C. auris* treated with chloroquine resulted in increased membrane fluidity due to reduced iron depletion that altered membrane sterol content and ultimately led to increased antifungal drug uptake [61,62].

Enhanced oxidative stress and the accumulation of ROS are recognized mechanisms through which natural compounds exert antifungal activity in *Candida* species [52,63,64]. Several antifungal compounds, including monoterpene phenols, retigeric acid B, and naphthoquinoidal compounds, have been shown to induce intracellular ROS production in *C. albicans* [65]. Interestingly, in the present study, *C. glabrata* exhibited a more sustained ROS response following AEA treatment compared with *C. auris* and *C. parapsilosis*, which showed only transient increases in ROS levels (Figure 6). This prolonged oxidative response may reflect species-specific differences in stress adaptation pathways, including distinct mitochondrial and lipid vulnerabilities that are specific to *C. glabrata* [66,67]. *C. glabrata* possesses a well-developed oxidative stress-response network involving catalases, superoxide dismutase, glutathione-associated pathways, and multiple stress-responsive transcription factors, which together coordinate adaptation to oxidative environments [68]. Transcriptomic studies have further demonstrated that oxidative stress in *C. glabrata* induces extensive temporal regulation of metabolic, membrane-associated, and transporter-related pathways [69]. In contrast, *C. parapsilosis* has been observed to actively diminish its oxidative stress response. This was observed after itraconazole treatment; itraconazole-induced ROS was abolished by an upregulation of antioxidant enzymes like catalase, which significantly abolishes ROS levels [70]. *C. auris*, as well, demonstrated robust stress response systems as well as robust mitochondrial integrity mechanisms and requires a more intensive trigger in order to produce dangerous ROS levels and keep a sustained ROS response [71,72].

Given the concurrent alterations in membrane fluidity, permeability, and membrane potential observed in the present study, the sustained ROS accumulation in *C. glabrata* may indicate prolonged mitochondrial and metabolic stress associated with maintaining cellular homeostasis following AEA-induced membrane perturbation. Thus, ROS accumulation may represent a downstream consequence of AEA-induced membrane and metabolic stress rather than the primary mechanism underlying its antifungal activity.

This study has several limitations, including the lack of in vivo testing, the use of laboratory reference strains rather than recent clinical outbreak isolates, and restriction to specific time points. To build on these single-agent findings, future studies should evaluate AEA in combination with a broader range of antifungal classes and assess their therapeutic potential in relevant infection models.

## 5. Conclusions

Taken together, the present study demonstrates that AEA exerts antifungal activity against NAC species through disruption of membrane-associated functions, including alterations in membrane fluidity, permeability, membrane potential, and ROS production, thereby perturbing cellular homeostasis. Importantly, these effects were species-dependent, highlighting fundamental differences in membrane organization, oxidative stress responses, and adaptive capacity among *C. auris*, *C. glabrata*, and *C. parapsilosis*. Collectively, these findings provide new insights into the antifungal activity of AEA and suggest that membrane-associated processes represent major targets of its action. The species-specific responses observed among NAC pathogens highlight the importance of membrane composition and stress adaptation pathways in determining susceptibility to AEA but need to be studied in more depth. These results support further investigation of AEA as a potential antifungal agent against clinically relevant NAC species.

## Figures and Tables

**Table 1:** MIC and MBIC values for *C. albicans*, *C. glabrata*, and *C. parapsilosis* strains.

Strain	MIC Fluconazole (µg/mL)	MIC AEA (µg/mL)	MBIC Fluconazole (µg/mL)	MBIC AEA (µg/mL)
*Candida albicans* 90028	1.56	>500	<1	>500
*Candida glabrata* 15126	6.25	32	<1	500
*Candida parapsilosis* 22019	1.56	8–31	<1	500

**Table 2:** MIC and MBIC values for *C. auris* strains.

Clinical Strain	Clade	MIC Fluconazole (µg/mL)	MIC AEA (µg/mL)	MBIC Fluconazole (µg/mL)	MBIC AEA (µg/mL)
B12896	I	>500	250	500	>500
MYA-5001(B112220)	II	8	62	8	250
MYA-5002(B112221)	III	125	250	125	250
B11247	IV	>500	>500	>500	>500
B12322	IV	>8	500	8	500
7115-A1	III	125	31	125	125
3089-A2	III	125	31	125	125

## Data Availability

Raw data for the figures are available upon reasonable request from the corresponding author.

## Abbreviations

The following abbreviations are used in this manuscript:

AEA	N-arachidonoyl ethanolamine, Anandamide
NAC	Non-*albicans Candida*
IC	Invasive candidiasis
MIC	Minimum inhibitory concentration
MBIC	Minimum biofilm inhibitory concentration
OD	Optical density
SDA	Sabouraud dextrose agar
MOPS	morpholinepropane-sulfonic acid
YPD	Yeast extract peptone dextrose
CFU	Colony forming units
PBS	Phosphate-buffered solution
CLSI	Clinical and Laboratory Standards Institute
MTT	3-(4,5-Dimethyl-2-thiazolyl)-2,5-diphenyl-2H-tetrazolium bromide
GP	General polarization
SDCM	Spinning disk confocal microscopy
PI	Propidium Iodide
DiS-C3(3)	3,3- dipropylthiacarbocyanine iodide
Req	Ratio equilibrium
ROS	Reactive oxygen species
DCFH-DA	2′,7′-dichlorofluorescein diacetate
R6G	Rhodamine 6G
MFS	Major facilitator superfamily
MDR	Mutlidrug resistance
ABC	ATP-binding cassettes
EM	Extracellular matrix
